# Exploration of the Hsa-miR-1587–Protein Interaction and the Inhibition to CASK

**DOI:** 10.3390/ijms221910716

**Published:** 2021-10-03

**Authors:** Lulu Zhang, Jiang Zhou, Ming Xu, Gu Yuan

**Affiliations:** 1 Research Center of Basic Medicine, Jinan Central Hospital Affiliated to Shandong First Medical University, Jinan 250013, China; zhanglulu@pku.edu.cn; 2 Beijing National Laboratory for Molecular Sciences, Department of Chemical Biology, College of Chemistry and Molecular Engineering, Peking University, Beijing 100871, China; guyuan@pku.edu.cn; 3 Key Laboratory of Cardiovascular Molecular Biology and Regulatory Peptides of Ministry of Health, Key Laboratory of Molecular Cardiovascular Sciences of Ministry of Education, Department of Cardiology, Institute of Vascular Medicine, Peking University Third Hospital, Bejing 100191, China; xuminghi@bjmu.edu.cn

**Keywords:** miR-1587, microRNA-protein interaction, CASK, G-quadruplex, regulation

## Abstract

Hsa-miR-1587 has been found to be capable of forming G-quadruplex structures and is overexpressed in multiple cancer cell lines. Here, we explored the interactions between miR-1587 and proteins. HuProt™ human proteome microarray was utilized to screen the binding proteins, and it was discovered that CASK could bind to miR-1587 on the base of the G-quadruplex structure. Moreover, reelin and p21, which are downstream of CASK, were downregulated both transcriptionally and translationally by miR-1587, uncovered by q-RT-PCR and Western blot assays. Bioinformatic analysis was performed on STRING and Panther platforms, leading to the discovery that miR-1587 may be involved in intracellular metabolic and transcriptional physiological processes. This study explores the interaction of hsa-miR-1587 with proteins and provides a new strategy for the regulation of G-rich microRNA’s function.

## 1. Introduction

MicroRNA (miRNA/miR) plays an important role in the normal physiological processes and abnormal pathology [1] of cells. Therefore, research on the relationship between microRNA and disease has enormous potential, including the regulation of cancer-related pathways, cell cycle control, and DNA damage. This investigation in the role of microRNA has already been involved in clinical studies and has confirmed its function as a critical biomarker for disease diagnosis and a target for cancer treatment. The earliest research on the relationship between microRNA and cancer was reported in chronic lymphocytic leukemia (CLL) [2], leading to the attention of related studies. MicroRNA expression is generally downregulated in tumors, and their different expression contribute to distinguishing normal and cancerous tissues, as well as identify the origin of tissues in some cancers [3,4].

The research regarding interactions between microRNA and mRNA has grown substantially over the previous two decades, and the studies of interactions between microRNA and protein have been developing gradually. Different binding proteins including the AGO family, have shown different binding properties to microRNA (miRBPs). The interaction between microRNA and protein can be divided into three classes. The first class is the competition mechanism. Some miRBPs compete with AGO2 for binding to microRNA, thereby hindering the functioning of microRNA [5,6,7]. Second is the transfer effect; miRBPs can also transmit microRNA to AGO2, which works together to complete microRNA-related regulation [5,8,9]. In addition, AGO2 binds to microRNA to regulate the activity of other genes, whereas, in fact, its own expression activity is regulated by microRNA in return. For example, miR-346 binds to the 3′-UTR of AGO2, which is mediated by GRSF protein, and could be identified as an activation effect [10]. In addition, Lin28 will recruit TUT4/PUP-2 to *let-7* precursors for mediating terminal uridine, thus preventing Dicer binding and *let-7* maturation, leading to microRNA synthesis blocking [11]. Another interesting model is the interaction between hnRNP E and miR-328 [12], which functions as an RNA decoy to interact with hnRNP E2 and modulate CEBPA finally.

A microRNA database (www.mirbase.org/index.shtml, accessed on 20 July 2021) was explored and more than 100 G-rich microRNAs were discovered, of which several are putative G-quadruplex sequences. A previous study reported that the mature G-rich sequence hsa-miR-1587 (UUGGGCUGGGCUGGGUUGGG) could fold into a G-quadruplex structure by ESI-MS, NMR, and CD spectroscopy [12], and is highly expressed in HeLa cells. Hsa-miR-1587 could be secreted through exosomes released by glioma-associated mesenchymal stem cells, and the downstream molecule NCOR1 was suppressed in glioma stem cells, leading to the enhancing proliferation, colony formation, and promoting tumor progression [13]. Despite the importance of hsa-miR-1587, its interaction with proteins has rarely been studied.

This study selected the G-rich hsa-miR-1587 as an example to probe the interactions between microRNA G-quadruplex and proteins, and CASK was found to be an has-miR-1587 target. CASK is a multi-domain scaffold protein that is essential for synaptic membrane protein anchoring and ion channel transport, and the interaction suggested an important regulation approach to microRNA. 

## 2. Results and Discussion

### 2.1. Different Structures of miR-1587 Bound to Different Proteins

10 OD FAM-labeled miR-1587 was divided into two portions, and the first solution was 30 mM Tris-HCl, 150 mM KCl, while the second was 30 mM Tris-HCl only, followed by annealing from 95 °C to 4 °C incrementally. According to a previous study [13], miR-1587 in the first condition formed a stable parallel strand G-quadruplex structure, while the miR-1587 in the second condition remained primarily unbound in its free form conformation. Hsa-miR-1587 with G-quadruplex and in its free form were incubated and eluted with two proteome chips, respectively, and proteins which bound to miR-1587 could be detected because of FAM function labeled on the microRNA sequence. Multiple studies have applied FAM for G-quadruplex labeling [14,15] since it has high sensitivity and does not influence the structure and result. Positive and negative spots were designed as control spots to confirm the microarray quality, and those control spots can be viewed in Figures S1 and S2. GenePix 4200A (Axon Instruments, Molecular Devices, San Jose, CA, USA) was utilized to detect the FAM function at the wavelength of 488 nm and obtain the raw data, followed by GenePix Pro-6.0 software analysis. The SNR values were calculated as previously described [16] and then sequenced to evaluate the binding intensity between miR-1587 and proteins. 

**Figure 1:**
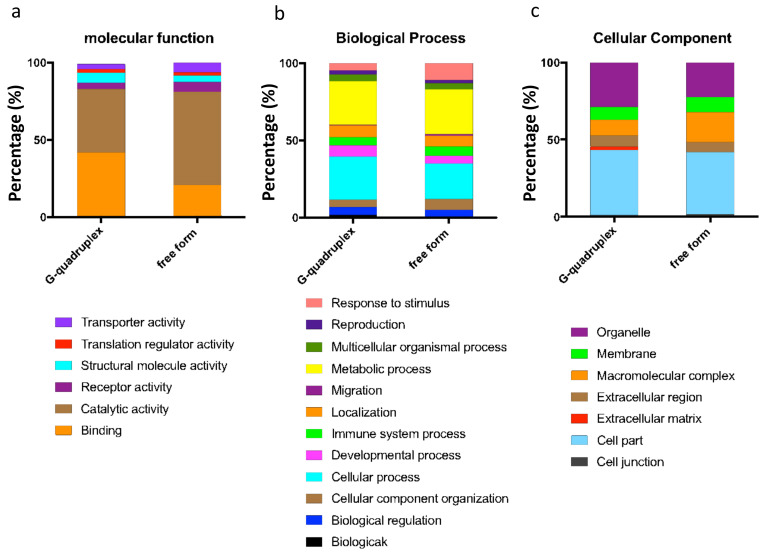
Panther analysis of the comparison between 182 kinds of G‒quadruplex binding proteins and 133 free from binding proteins in (**a**) Molecular Function, (**b**) Biological Process, and (**c**) Cellular Component. In the molecular function classification, the binding-related protein in the chip of free-form miR-1587 decreased, and the protein with catalytic activity increased, which showed obvious differences. In the biological process classification, the cellular process proteins in the chip of free-form miR-1587 decreased while the response to stimulus-related protein displayed obvious improvement. In the cellular component classification, both cell part and organelle-related protein decreased, while macromolecular complex related molecules increased.

Figure S1 showed the chip containing the G-quadruplex structure and the sorted 47617 SNR data, with a cutoff value of 3. One hundred and eighty-two kinds of proteins were revealed to belong to the top 1% of the total protein number, 19394. Therefore the 182 kinds of proteins were shown to be miR-1587 G-quadruplex binding proteins (Table S1). In contrast, for the chip containing free-form miR-1587, the cutoff value was set to be 1.30, and the first 133 proteins were obtained, as shown in Figure S2 and Table S2. The binding proteins were different according to different miR-1587 structures, and Figure 1 showed the classification difference.

In the molecular function classification, the binding-related protein in the chip of free-form miR-1587 decreased by 20% compared to the chip of the G-quadruplex structure. In addition, the protein with catalytic activity increased to 60.4%, which showed obvious differences. The primary difference caused by the two conditions was the miR-1587 structure. Based on the results of the experiment, we can infer that different microRNA can bind to different proteins and that the secondary structure can influence the interactions between microRNAs and proteins. Here are some examples to better understand our results. Nuclear factor NF-κB is a well-known transcription factor present in most cells and is responsible for a series of signal transduction. Our results showed that miR-1587 in the G-quadruplex structure can bind to NF-κB while free-form miR-1587 could not. In addition, free-form miR-1587 could bind to WNT6, which is a transmembrane receptor related to gastric cancer, while the G-quadruplex could not. Hence, the different structures showed different binding properties with important regulators in cells, suggesting the miR-1587 may modulate cell processes through its structure.

According to the biological process and cellular component analysis, the proteins which ranked at the front of the list are different, although not as obvious as in molecular functional classification. The results provide supplementary support for the role the structure plays in microRNA–protein interactions.

The STRING platform helps in analyzing the molecular function of proteins. Figure S3 showed the binding protein’s functional network from miR-1587 in the G-quadruplex and free-form structure. When miR-1587 was present as a G-quadruplex conformation, the binding protein was more dispersed on the long-term functional line, on the other hand, free-form miR-1587 binding proteins were discrete and no obvious functional pathway was found. Neither of the STRING analyses had shown a concentrated protein cluster in the previous study [16], which may be related to the interaction mechanism. Furthermore, there was a big difference between the two assays in molecular function. The abundance of binding proteins and catalytic active proteins were both estimated to be 40% in the G-quadruplex assay, however, the abundance of binding proteins decreased to about 20% and catalytic active proteins increased to about 60% in the free-form miR-1587 assay, indicating that the different secondary structures prefer to bind to different proteins. In addition, for many specific proteins, the binding SNRs between the two assays were significantly different. For example, CASK ranked 10th in the G-quadruplex miR-1587 assay while ranked about 2000th in the free-form miR-1587 assay, and Supt6 ranked 17th in the G-quadruplex miR-1587 assay while ranked over 2000th in the free-form miR-1587 assay, respectively, indicating G-quadruplex formation benefited microRNA and protein interaction for these proteins. In contrast, some proteins exhibited higher SNRs in the free-form miR-1587 assay compared to the G-quadruplex miR-1587 assay, such as CCDC130, NAA16, BIRC5, implying the formation of G-quadruplex sometimes blocked microRNA and protein interactions.

### 2.2. miR-1587 in G-Quadruplex Structure Bound to CASK

By contrasting the SNR values between the two assays, the binding properties of different proteins could be analyzed. CASK was found to bind to miR-1587 in the G-quadruplex structure with a SNR of 8.75, which was ranked 10th, belonging to 0.1% in the whole microarray assay proteome and indicating strong binding affinity between the CASK protein and miR-1587 G-quadruplex. In contrast, the binding SNR with free-form miR-1587 was 1.07, implying similar signal intensity with background, which could be considered as no combination.

HuProt™ human proteome microarray assay has been performed for RNA–protein interactions in multiple studies. Different RNA probes, which could be considered as random control samples, were used for RNA-binding protein (RBP) screening [17,18,19], and CASK was not selected as potential RBP in any of these studies, consistent with our hypothesis that the interaction was based on microRNA G-quadruplex rather than RNA nucleotides.

**Figure 2:**
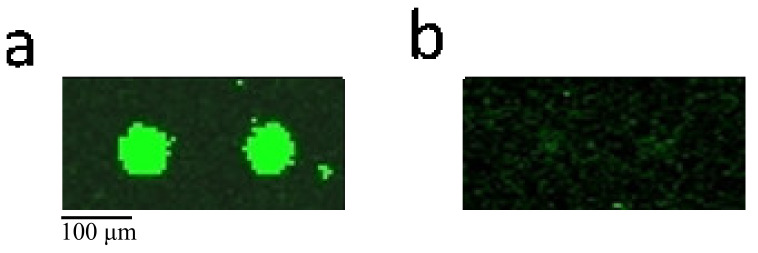
The imaging results of CASK in (**a**) chip of miR-1587 in the G-quadruplex structure (**b**) chip of miR-1587 in free form. The miR-1587 was labeled by FAM and the proteins were fixed on the surface of chips, leading to the acknowledgment that the fluorescence signals indicated the binding between miR-1587 and the protein in a specific area. This figure indicates that CASK could bind to the miR-1587 in the G-quadruplex structure but could not bind to miR-1587 in free form.

CASK regulates neurodevelopment and gene expression, and it also binds to cell surface proteins, including myeloid precursor proteins, neurexins, and syndecans. The association between the extracellular matrix and the actin cytoskeleton can be mediated by the interaction between CASK and syndecan and actin/hemagglutinin binding proteins. CASK protein was selected as a research target because of its important function in cells. Figure 2 shows the comparison between the CASK imaging results from two chips.

As the main bearer of biological function, proteins play an important role in various life activities and are important targets for disease treatment. With the development of experimental technology, the structural resources of biological macromolecules and ligands have been gradually enriched. The evolution of computer technology in recent decades has made it possible for molecular simulation docking. The advantages of rationality and efficiency help to reduce the blindness of experimental research and accelerate the research development, thus the computer simulation method was performed to investigate the binding pattern between miR-1587 G-quadruplex and CASK.

ESI-MS and CD results provided evidence that miR-1587 formed a three-layer parallel G-quadruplex structure in vitro, and RNaseT1 footprinting experiment had been used to determine the bases involved in G-quadruplex, therefore, the three-dimensional structure of miR-1587 G-quadruplex could be simulated by molecular dynamics.

More than two thousand kinds of known human protein structures have been used to establish a protein library, and miR-1587 G-quadruplex was simulated to dock with these proteins, respectively. Finally, less than ten kinds of combinations were selected including CASK.

**Figure 3:**
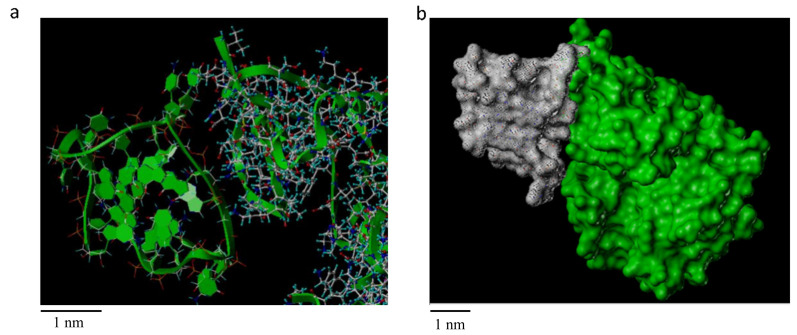
Computer simulation result of miR-1587 G-quadruplex and CASK docking in (**a**) ribbon pattern (**b**) surface pattern. As shown in (**a**), the G-quadruplex structure (left part, green) could bind to the CASK side of the α-helix and β-sheet, and (**b**) shows the G-quadruplex formed by miR-1587 had a surface similarity to CASK, possibly through multiple positions of bonding.

The docking result from AutoDock4 is shown in Figure 3, and Figure 3a displays the CASK skeleton in ribbon pattern, while Figure 3b shows the overall structure in surface pattern to clarify the structure of the embedded interaction. It was obvious that the side of the G-quadruplex formed by miR-1587 had a surface similarity to the side of the α-helix and β-sheet of the CASK protein, thus multiple positions of bonding were available, suggesting the binding could remain stable.

The docking result of other proteins with miR-1587 is shown in Figure S4, which displayed seven proteins that were receivable for docking. Based on the surface of the protein and the amino acid type, the proteins had different docking patterns with miR-1587 G-quadruplex, which might help further research.

### 2.3. miR-1587 G-Quadruplex Influenced CASK Downstream Genes Reelin and p21

The protein microarray assay and computer simulations indicated that miR-1587 could bind to CASK based on the G-quadruplex structure. Furthermore, the downstream changes caused by miR-1587 were verified via q-RT-PCR and Western blot.

**Figure 4:**
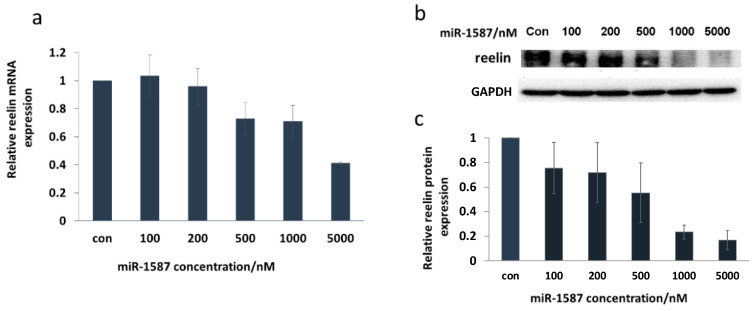
Reelin was down-regulated by miR‒1587 in HeLa cells. (**a**) q-RT-PCR results for reelin, with β-actin as a control. (**b**) Western blot of reelin, with GAPDH as a control. (**c**) Histogram of reelin protein levels at different miR‒1587 concentrations. Data were obtained through two independent sets of HeLa cells and the regulation results of each set were applied for analysis.

Reelin is a downstream gene of CASK [20], and recently, CASK and reelin were found to be overexpressed in human esophageal cancer [21], transcriptionally and translationally, leading to a deep exploration of them in disease research. It was found that the mRNA and protein levels of reelin were downregulated after miR-1587 transfection, as shown in Figure 4.

Figure 4a shows the q-RT-PCR result, with β-actin as a control, and it was clear that the mRNA level of reelin decreased with the increase in miR-1587 concentration. The transcription level declined to about 40% at 5 μM of miR-1587, indicating obvious inhibition. Figure 4b displays the Western blot assay result, and Figure 4c shows the histogram of the Western blot imaging result, demonstrating that protein expression decreased which was consistent with the transcription inhibition. The q-RT-PCR experiment result suggested that the binding between miR-1587 and CASK could influence the downstream gene reelin and showed effective inhibition.

P21, which is another downstream target of CASK, has multiple biological functions, including mediating G1 arrest [22] and regulating cell apoptosis [23,24]. Rongju [25] found that overexpression of human CASK resulted in a decrease in cell growth rate, and then confirmed that CASK modulated p21 mRNA and protein levels and activates p21 expression in a time-dependent manner, therefore, CASK and p21 could be regarded as regulation targets.

**Figure 5:**
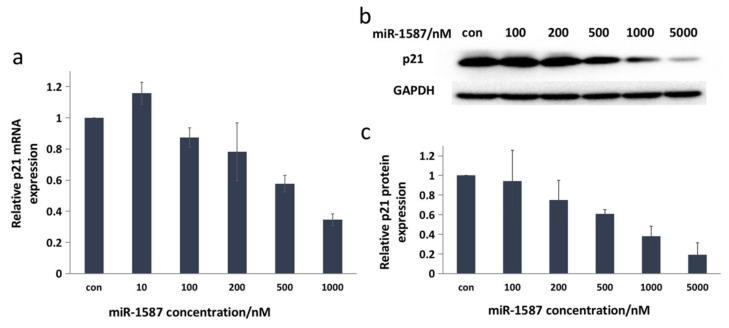
P21 was downregulated. (**a**) q-RT-PCR results for p21, with beta-actin as a control. (**b**) Western blot of p21, with GAPDH as a control. (**c**) Histogram of p21 protein levels at different miR‒1587 concentrations. Data were obtained through two independent sets of HeLa cells and the regulation results of each set were applied for analysis.

The q-RT-PCR assay showed that the mRNA expression of p21 decreased with the miR-1587 concentration increasing and changed to about 35% at 1 μM of miR-1587, as shown in Figure 5a. In addition, Figure 5b displays the Western blot result and it was obvious that p21 protein expression was downregulated with the increase in miR-1587 concentration. According to the quantitative results shown in Figure 5c, the translation level declined to about 38% when the concentration of miR-1587 was 1 μM, which was consistent with the q-RT-PCR result, indicating that miR-1587 could inhibit CASK downstream proteins.

A similar regulation trend was observed (Figure S5) within 293T and A549 cell lines using the Western blot assay, indicating the regulation of miR-1587 and CASK could function in multiple cell lines.

**Figure 6:**
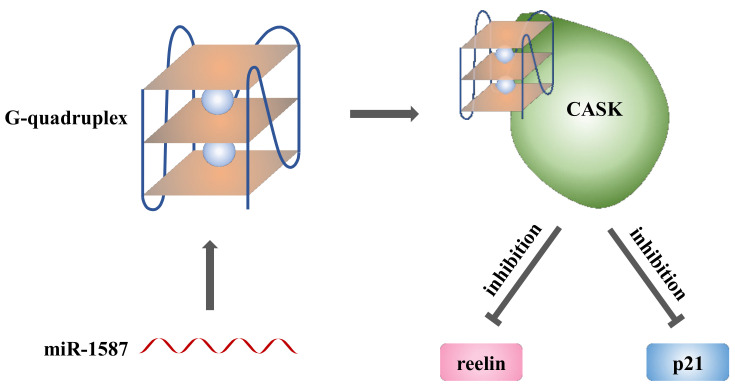
Scheme of miR-1587 regulation. Mature miR-1587 could bind to CASK protein in G-quadruplex form, and the binding could influence CASK downstream gene reelin and p21. It might even influence intracellular signaling pathways, which would be a new regulatory approach.

Based on the investigation, we could draw the miR-1587 regulation scheme, as shown in Figure 6. Mature miR-1587 of the G-quadruplex form could bind to the CASK protein, leading to downregulation of CASK downstream gene reelin and p21, which might even influence intracellular signaling pathways, which would be a new regulatory approach.

## 3. Materials and Methods

### 3.1. Materials

The miR-1587 sample and PCR primers were purchased from TaKaRa Biotechnology (Dalian, China) and RuiBiotech (Beijing, China). Anti-reelin and anti-p21 antibodies were purchased from Abcam (Cambridge, UK), ABclonal (Wuhan, China), and Beyotime (Shanghai, China). Lipo2000 was purchased from Invitrogen (Thermo Fisher Scientific, San Francisco, CA, USA). DMEM (Corning, Glendale, AZ, USA), 10% fetal bovine serum (CellMax, Sunnyvale, CA, USA), Opti-MEM (Gibco, Thermo Fisher Scientific, San Francisco, CA, USA), 1% penicillin-streptomycin (Gibco, Thermo Fisher Scientific, San Francisco, CA, USA) were used for cell culture.

### 3.2. Human Proteome Microarray Assay

A human proteome microarray assay was carried out on HuProt™ microarray (CDI, Mayaguez, PR, USA). FAM-labeled miR-1587 (10 OD) was divided into two portions, each containing 3 mL of sample, with a final concentration of 7.8 μM. The first solution was 30 mM Tris-HCl, 150 mM KCl and the second solution was 30 mM Tris-HCl. Both samples were cooled incrementally from 95 °C to 4 °C.

The proteome microarray was carried out as previously described [16]. Proteome microarrays were blocked with blocking buffer (1% BSA in 0.1% Tween-20; TBST) for 1 h at room temperature with gentle shaking. MiR-1587 samples were incubated on the proteome microarray at room temperature for 1 h. The microarrays were washed with TBST three times for 5 min in each washing. The microarrays were spun dry at 250 g for 3 min and were scanned at 488 nm with a GenePix 4200A microarray scanner (Axon Instruments, Molecular Devices, San Jose, CA, USA) to visualize and record the results. GenePix Pro-6.0 software (Axon Instruments, Molecular Devices, San Jose, CA, USA) was used for data analysis. This experiment was accomplished in Shengce Tao’s Lab (Shanghai Center for Systems Biomedicine, Shanghai, China) with help from Chengxi Liu.

### 3.3. Computer Simulation

The PDB files on the human protein from RCSB Protein Data Bank were examined by SYBYL 7.0 software (Tripos Inc., St. Louis, MO, USA) to remove all the ligands and examined the integrity of sequences. If the structure was incomplete, the SWISS-MODEL platform was used to reconstruct a complete sequence and three-dimensional structure information of the protein, and 1231 kinds of protein files were modified in total. The molecular docking (AutoDock4 [26] in DOVIS 2.0 [27]) was carried out between the miR-1587 G-quadruplex structure and all the protein structures. The docking results were analyzed and evaluated one by one to increase reliability.

### 3.4. Quantitative Real-Time PCR

All cells were cultured in 5% CO_2_ at 37 °C. No more than 5 × 10^5^ HeLa/293T/A549 cells were plated in each well of the 6-well plates, and no more than 1 × 10^5^ cells were plated in each well of the 12-well plates. Cells were fasted in DMEM without FBS or penicillin-streptomycin for 4 h after adherence, followed by transfection of miR-1587 through Lipo2000. We diluted Lipo2000 reagent and miR-1587 of different concentrations in Opti-MEM and incubated for 15 min at room temperature after mixing both parts, then added the mixture into DMEM medium without FBS or penicillin-streptomycin. The final concentration of Opti-MEM was 50%, the final concentration of Lipo was 15 μL/mL while miR-1587 concentrations were 0, 10 nM, 100 nM, 200 nM, 500 nM, 1 μM, and 5 μM in HeLa sets, respectively. The transfection was kept for 6 h and then the medium was replaced by DMEM with 10% FBS and 1% penicillin-streptomycin and cells were harvested at 24 h. Two independent sets of HeLa cells were employed for Western and PCR, and three independent sets (0, 0.1 nM, 1 nM, 10 nM) were designed for 293T and A549 cell lines.

Total RNA was extracted using TRIzol reagent (Xinjingke, Beijing, China) and was measured using a NanoDrop 1000 (Thermo Scientific, San Jose, CA, USA). Then, 1 μg of total RNA was used for cDNA synthesis by TransScript II All-in-One First-Strand cDNA Synthesis SuperMix for qPCR Kit (#AH341, TransGen Biotech, Beijing, China) in a volume of 20 μL at 55 °C for 15 min and at 85 °C for 5 s. Next, the product was diluted to a final volume of 200 μL in preparation for PCR. Quantitative real-time PCR (q-RT-PCR) reactions were carried out in a volume of 20 μL containing TransStart Tip Green qPCR SuperMix (#AQ141, TransGen Biotech, Beijing, China), 0.2 μM of forward and reverse gene-specific primers, and 5 μL of cDNA. An Eppendorf Realplex Real-time PCR System was used to detect the optical reactions of the target gene, β-actin was used as a control. The q-RT-PCR was conducted at 94 °C for 2 min, followed by 40 cycles of 94 °C for 5 s, 55 °C for 15 s, and 72 °C for 10 s; melting curves were measured to confirm the amplification specificity.

Primer sequence information: Reelin-F (5′-CAACCCCACCTACTACGTTCC-3′), Reelin-R (5′-TCACCAGCAAGCCGTCAAAAA-3′), p21-F (5′- TGTCCGTCAGAACCCATGC-3′), p21-R (5′-AAAGTCGAAGTTCCATCGCTC-3′), beta-actin-F (5′-TTTTGGCTATACCCTACTGGCA-3′), beta-actin-R (5′- CTGCACAGTCGTCAGCATATC-3′).

### 3.5. Western Blot

The cell culture, transfection, and incubation were the same as described in the Section 3.4. The cells were then lysed in lysis buffer, and a protein solution was obtained after centrifugation and boiling for 5 min at 95 °C. Total protein of each sample was separated by 8% sodium dodecyl sulfate-polyacrylamide gel electrophoresis (SDS-PAGE). The samples were then transferred onto 0.45 μm PVDF membranes at 300 mA for 90 min. After transferring, the membrane was blocked with 5% non-fat milk for 0.5 h at room temperature (RT), and then membranes were incubated with primary antibodies (Reelin 1:1000, p21 1:1000, GAPDH 1:2000) at 4 °C overnight, and then for 0.5 h with secondary antibodies at 25 °C. The membranes were washed with PBST after every incubation. The Immobilon Western Kit (Millipore, Billerica, MA, USA) was used for chemiluminescent detection, and ImageJ was used for quantitative analysis.

### 3.6. Bioinformatic Analysis

In this paper, DAVID (https://david.abcc.ncifcrf.gov, 29 May 2016) was employed to convert formats of gene lists into gene IDs, which could be better recognized by other tools such as STRING or Panther. In the “Multiple proteins” part, after importing proteins’ names and defining the organism as homo sapiens, STRING (https://www.string-db.org, 22 May 2016) could recognize the assigned protein while ignoring the nonassigned ones. STRING also provided the interaction pattern within the imported protein list, and the pattern could be modified by removing some isolated molecules, which helped us focus on the main functional interactions. Then, we applied the gene ID or name into Panther analysis (http://www.pantherdb.org/, 5 June 2016), which could provide the functional classification of imported genes. 

## 4. Conclusions

In this study, we carried out the HuProt™ human proteome microarray assay and confirmed that binding protein populations are different according to different miR-1587 structures. In particular, CASK could bind miR-1587 G-quadruplex and computer simulations displayed the docking pattern. Furthermore, CASK downstream genes reelin and p21 were downregulated by miR-1587 both transcriptionally and translationally. This research investigated the interaction between miR-1587 G-quadruplex and proteins, especially CASK, and offers new possibilities for exploring G-rich microRNA.

## Data Availability

All data are contained within this manuscript.

## Supplementary Materials

Supplementary Materials are available online at https://www.mdpi.com/article/10.3390/ijms221910716/s1.

## Abbreviations

miRNA/miR	MicroRNA
CASK	Calcium/Calmodulin-Dependent Serine Protein Kinase
CLL	Chronic Lymphocytic Leukemia
AGO	Archipelago
miRBP	MicroRNA Binding Protein
3′-UTR	3′-Untranslated Regions
GRSF	Guanine-Rich Sequence Factor
TUT4	Terminal Uridylyltransferase 4
PUP-2	PAP-Associated Domain-Containing Protein
hnRNP	Heterogeneous Nuclear Ribonucleoprotein
CEBPA	CCAAT Enhancer Binding Protein Alpha
ESI-MS	Electrospray Ionization Mass Spectrometry
CD	Circular Dichroism
NMR	Nuclear Magnetic Resonance
NCOR1	Nuclear Receptor Corepressor 1
q-RT-PCR	Quantitative Real-Time Polymerase Chain Reaction
BSA	Bovine Serum Albumin
TBST	Tris Buffered Saline with Tween
DMEM	Dulbecco’s Modified Eagle’s Medium
FBS	Fetal Bovine Serum
SDS-PAGE	Sodium Dodecyl Sulfate-Polyacrylamide Gel Electrophoresis
GAPDH	Glyceraldehyde-3-Phosphate Dehydrogenase
PBST	Phosphate-Buffered Saline with Tween
SNR	Signal to Noise Ratio
NF-κB	Nuclear Factor kappa B
WNT6	Wingless-Type MMTV Integration Site Family, member 6
CCDC130	Coiled-Coil Domain Containing 130
NAA16	N-Alpha-Acetyltransferase 16, NatA auxiliary subunit
BIRC5	Baculoviral IAP Repeat Containing 5

## References

[1] Ambros V. (2004). The functions of animal microRNAs. Nature.

[2] Calin G.A., Sevignani C., Dan Dumitru C., Hyslop T., Noch E., Yendamuri S., Shimizu M., Rattan S., Bullrich F., Negrini M. (2004). Human microRNA genes are frequently located at fragile sites and genomic regions involved in cancers. Proc. Natl. Acad. Sci. USA.

[3] Blenkiron C., Goldstein L.D., Thorne N.P., Spiteri I., Chin S.-F., Dunning M.J., Barbosa-Morais N.L., Teschendorff A.E., Green A.R., Ellis I.O. (2007). MicroRNA expression profiling of human breast cancer identifies new markers of tumor subtype. Genome Biol..

[4] Sempere L.F., Christensen M., Silahtaroglu A., Bak M., Heath C.V., Schwartz G., Wells W., Kauppinen S., Cole C.N. (2007). Altered microRNA expression confined to specific epithelial cell Subpopulations in breast cancer. Cancer Res..

[5] Mukherjee K., Ghoshal B., Ghosh S., Chakrabarty Y., Shwetha S., Das S., Bhattacharyya S.N. (2016). Reversible HuR-microRNA binding controls extracellular export of miR-122 and augments stress response. EMBO Rep..

[6] Poria D.K., Guha A., Nandi I., Ray P.S. (2016). RNA-binding protein HuR sequesters microRNA-21 to prevent translation repression of proinflammatory tumor suppressor gene programmed cell death 4. Oncogene.

[7] Young L.E., Moore A.E., Sokol L., Meisner-Kober N., Dixon D.A. (2012). The mRNA Stability Factor HuR Inhibits MicroRNA-16 Targeting of COX-2. Mol. Cancer Res..

[8] Yoon J.H., Jo M.H., White E.J.F., De S., Hafner M., Zucconi B.E., Abdelmohsen K., Martindale J.L., Yang X.L., Wood W.H. (2015). AUF1 promotes let-7b loading on Argonaute 2. Genes Dev..

[9] Mourelatos Z., Dostie J., Paushkin S., Sharma A., Charroux B., Abel L., Rappsilber J., Mann M., Dreyfuss G. (2002). miRNPs: A novel class of ribonucleoproteins containing numerous microRNAs. Genes Dev..

[10] Guo J., Lv J., Liu M., Tang H. (2015). miR-346 Up-regulates Argonaute 2 (AGO2) Protein Expression to Augment the Activity of Other MicroRNAs (miRNAs) and Contributes to Cervical Cancer Cell Malignancy. J. Biol. Chem..

[11] Viswanathan S.R., Daley G.Q. (2010). Lin28: A MicroRNA Regulator with a Macro Role. Cell.

[12] Eiring A.M., Harb J.G., Neviani P., Garton C., Oaks J.J., Spizzo R., Liu S.J., Schwind S., Santhanam R., Hickey C.J. (2010). miR-328 Functions as an RNA Decoy to Modulate hnRNP E2 Regulation of mRNA Translation in Leukemic Blasts. Cell.

[13] Figueroa J., Phillips L.M., Shahar T., Hossain A., Gumin J., Kim H., Bean A.J., Calin G.A., Fueyo J., Walters E.T. (2017). Exosomes from Glioma-Associated Mesenchymal Stem Cells Increase the Tumorigenicity of Glioma Stem-like Cells via Transfer of miR-1587. Cancer Res..

[14] Chen H., Long H., Cui X., Zhou J., Xu M., Yuan G. (2014). Exploring the Formation and Recognition of an Important G-Quadruplex in a HIF1 alpha Promoter and Its Transcriptional Inhibition by a Benzo c phenanthridine Derivative. J. Am. Chem. Soc..

[15] Su R., Zheng H., Dong S., Sun R., Qiao S., Sun H., Ma X., Zhang T., Sun C. (2019). Facile detection of melamine by a FAM-aptamer-G-quadruplex construct. Anal. Bioanal. Chem..

[16] Zhang H.-N., Yang L., Ling J.-Y., Czajkowsky D.M., Wang J.-F., Zhang X.-W., Zhou Y.-M., Ge F., Yang M.-K., Xiong Q. (2015). Systematic identification of arsenic-binding proteins reveals that hexokinase-2 is inhibited by arsenic. Proc. Natl. Acad. Sci. USA.

[17] Liu L.C., Li T., Song G., He Q.X., Yin Y.F., Lu J.Y.Y., Bi X.J., Wang K.L., Luo S., Chen Y.S. (2019). Insight into novel RNA-binding activities via large-scale analysis of lncRNA-bound proteome and IDH1-bound transcriptome. Nucleic Acids Res..

[18] Barry G., Briggs J.A., Vanichkina D.P., Poth E.M., Beveridge N.J., Ratnu V.S., Nayler S.P., Nones K., Hu J., Bredy T.W. (2014). The long non-coding RNA Gomafu is acutely regulated in response to neuronal activation and involved in schizophrenia-associated alternative splicing. Mol. Psychiatry.

[19] Kang C.L., Qi B., Cai Q.Q., Fu L., Yang Y., Tang C., Zhu P., Chen Q.W., Pan J., Chen M.H. (2019). LncRNA AY promotes hepatocellular carcinoma metastasis by stimulating ITGAV transcription. Theranostics.

[20] Hsueh Y.P., Wang T.F., Yang F.C., Sheng M. (2000). Nuclear translocation and transcription regulation by the membrane-associated guanylate kinase CASK/LIN-2. Nature.

[21] Wang Q., Lu J.Y., Yang C.H., Wang X.Q., Cheng L., Hu G.X., Sun Y.T., Zhang X., Wu M., Liu Z.H. (2002). CASK and its target gene Reelin were co-upregulated in human esophageal carcinoma. Cancer Lett..

[22] Brugarolas J., Moberg K., Boyd S.D., Taya Y., Jacks T., Lees J.A. (1999). Inhibition of cyclin-dependent kinase 2 by p21 is necessary for retinoblastoma protein-mediated G(1) arrest after gamma-irradiation. Proc. Natl. Acad. Sci. USA.

[23] Huang W.S., Kuo Y.H., Kuo H.C., Hsieh M.C., Huang C.Y., Lee K.C., Lee K.F., Shen C.H., Tung S.Y., Teng C.C. (2017). CIL-102-Induced Cell Cycle Arrest and Apoptosis in Colorectal Cancer Cells via Upregulation of p21 and GADD45. PLoS ONE.

[24] Ropponen K.M., Kellokoski J.K., Lipponen P.K., Pietilainen T., Eskelinen M.J., Alhava E.M., Kosma V.M. (1999). P21/WAF1 expression in human colorectal carcinoma: Association with p53, transcription factor AP-2 and prognosis. Br. J. Cancer.

[25] Sun R.J., Su Y.Y., Zhao X.D., Qi J., Luo X.F., Yang Z.C., Yao Y.M., Luo X.D., Xia Z.F. (2009). Human calcium/calmodulin-dependent serine protein kinase regulates the expression of p21 via the E2A transcription factor. Biochem. J..

[26] Morris G.M., Huey R., Lindstrom W., Sanner M.F., Belew R.K., Goodsell D.S., Olson A.J. (2009). AutoDock4 and AutoDockTools4: Automated docking with selective receptor flexibility. J. Comput. Chem..

[27] Jiang X., Kumar K., Hu X., Wallqvist A., Reifman J. (2008). DOVIS 2.0: An efficient and easy to use parallel virtual screening tool based on AutoDock 4.0. Chem. Cent. J..

