# Supplementary material for: Exploration of the Hsa-miR-1587–Protein Interaction and the Inhibition to CASK
Source: Int J Mol Sci. 2021 Oct 3;22(19):10716. doi: 10.3390/ijms221910716 (PMC8509502; doi:10.3390/ijms221910716)
Supplement: Supplementary file 1 [file ijms-22-10716-s001.zip › ijms-1367394-SI.pdf]

Supporting Information

Exploration of the hsa-miR-1587-protein interaction and the inhibition to CASK

Lulu Zhang [1, 2], Jiang Zhou [2,*], Ming Xu [3] and Gu Yuan [2]

[1] Research Center of Basic Medicine, Jinan Central Hospital affiliated to Shandong First Medical University, Jinan 250013, China; zhanglulu@pku.edu.cn

[2] Beijing National Laboratory for Molecular Sciences, Department of Chemical Biology, College of Chemistry and Molecular Engineering, Peking University, Beijing 100871, China; guyuan@pku.edu.cn

[3] Key Laboratory of Cardiovascular Molecular Biology and Regulatory Peptides of Ministry of Health, Key Laboratory of Molecular Cardiovascular Sciences of Ministry of Education, Department of Cardiology, Institute of Vascular Medicine, Peking University Third Hospital, Bejing 100191, China; xuminghi@bjmu.edu.cn

* Correspondence: zhoujiang@pku.edu.cn

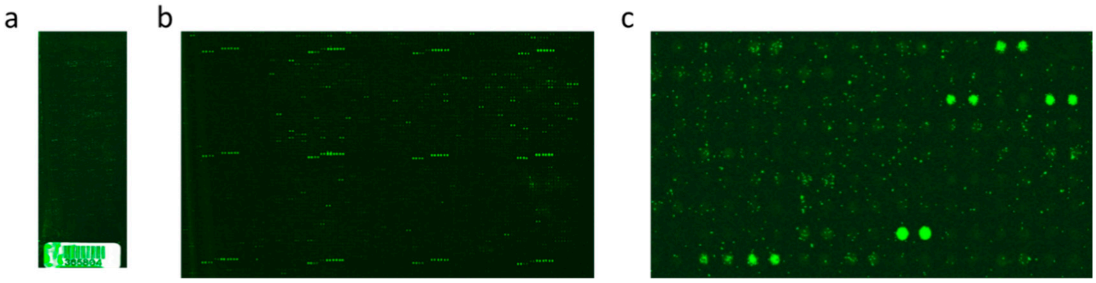

Figure S1. The imaging results of human proteome microarray assay with miR-1587 G-quadruplex structure. (a) overview of whole microarray assay (b) different blocks which contain control samples to indicate quality and parallelism (c) enlarged detail of microarray assay, duplicate for each protein.

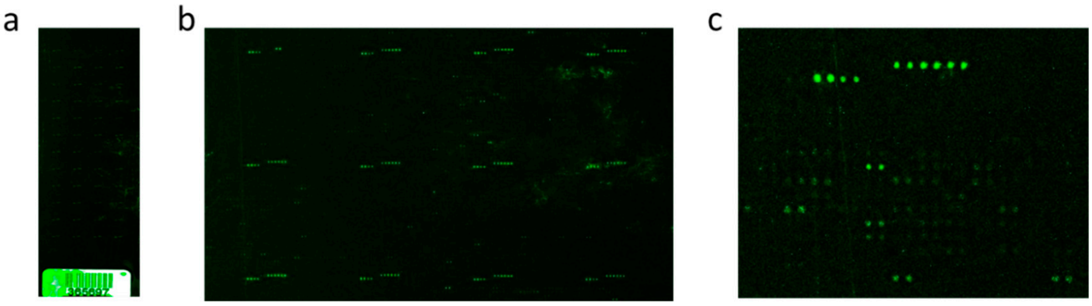

Figure S2. The imaging results of human proteome microarray assay with miR-1587 free form structure. (a) overview of whole microarray assay (b) different blocks which contain control samples to indicate quality and parallelism (c) enlarged detail of microarray assay, duplicate for each protein.

a

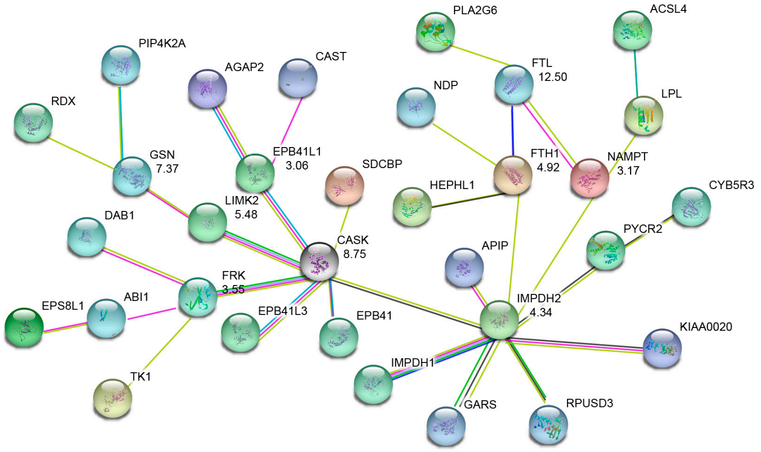

b

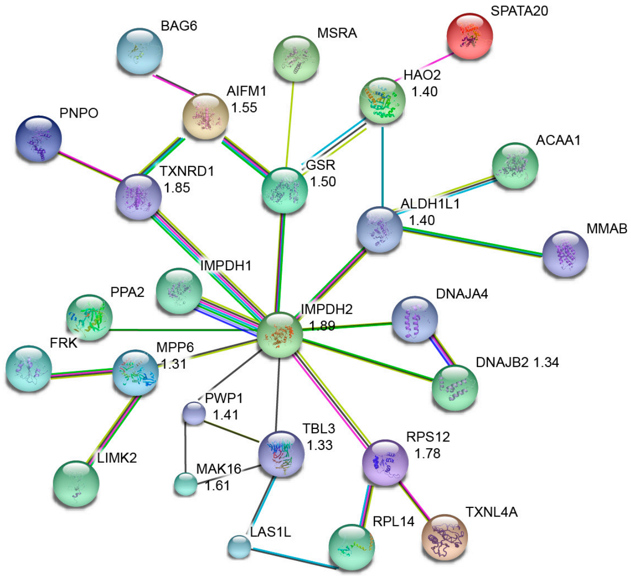

Figure S3. STRING analysis of binding proteins. (a) miR-1587 G-quadruplex binding proteins' enriched functional network (b) free form miR-1587 binding proteins' enriched functional network. Even though there were 182 and 133 binding proteins were considered to be strong binding proteins, most of them could not join into the same interaction network. After removing the isolated molecules, the left binding proteins were kept in the tightly connected network. As shown in (a), the patten displayed the network of miR-1587 G-quadruplex binding proteins. With FTL, CASK and IMPDH2 as nodes, there were many proteins that could interact with them, and these proteins were mainly concentrated in the functional processes related to binding, such as nucleic acid binding, heterocyclic compound binding, circular organic compound binding, etc. In contrast, the free form miR-1587 binding protein network was centered on IMPDH2, ALDH1L1 and GSR, and less members were recruited compared to miR-1587 G-quadruplex binding proteins. In conclusion, different miR-1587 structures could bind to different proteins and they may exhibit different influences and functions in cells.

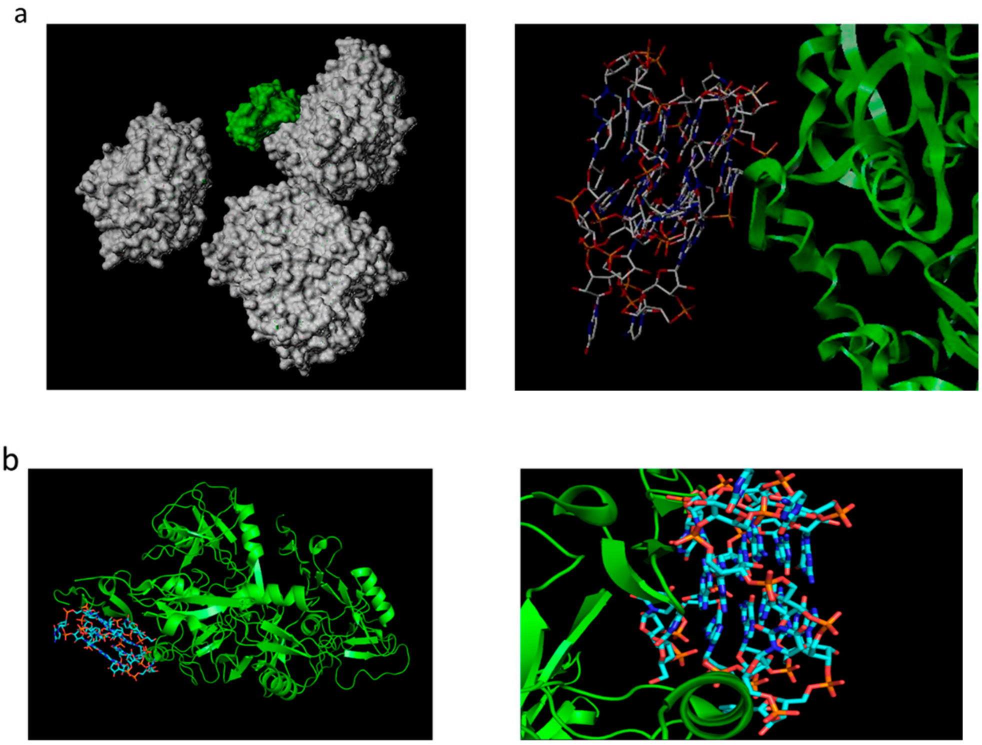

c

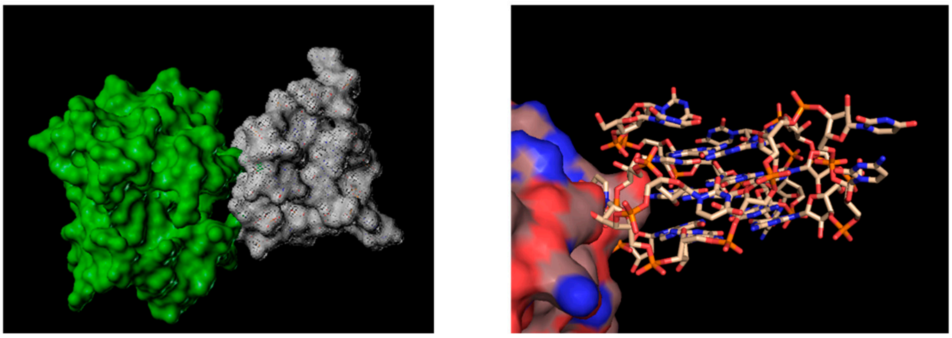

d

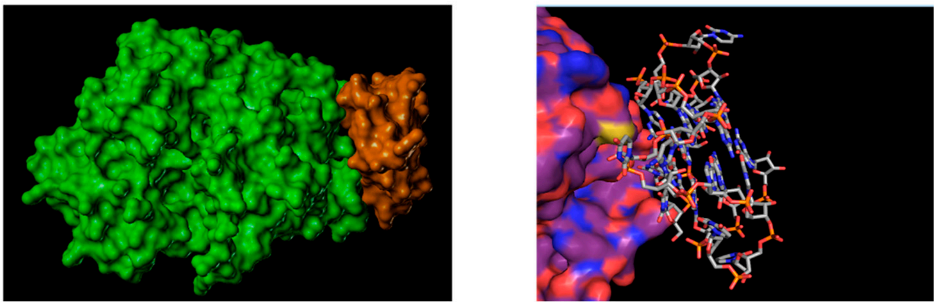

e

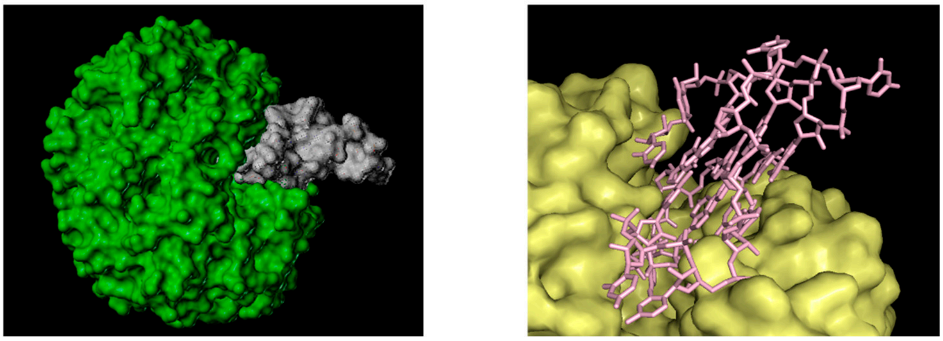

f

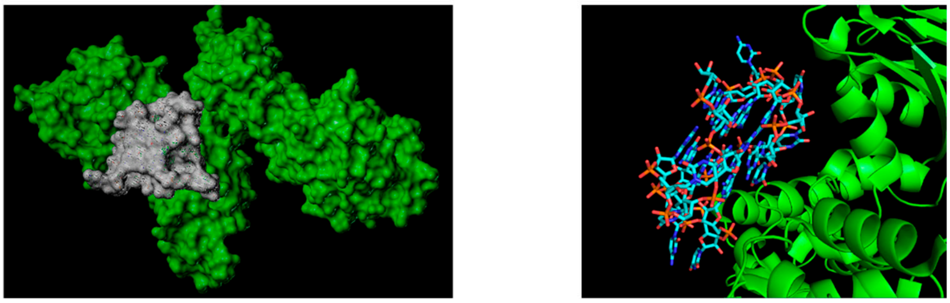

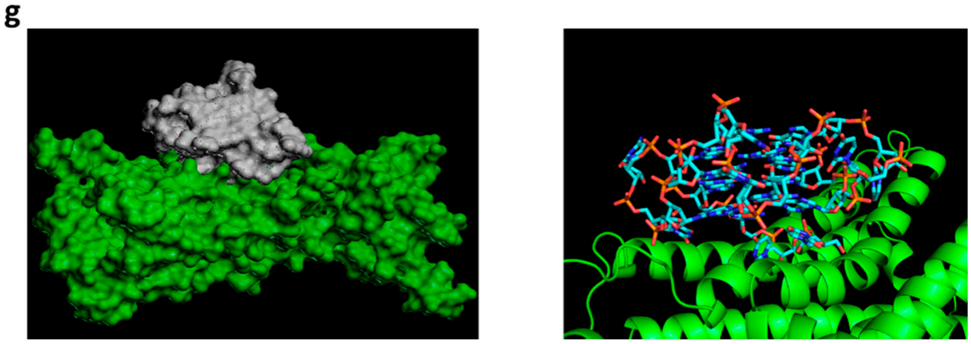

Figure S4. Computer simulation result of miR-1587 G-quadruplex and seven proteins. Left pictures: overview of proteins and miR-1587 G-quadruplex, right pictures: binding details between proteins and G-quadruplex. (a) P22830, ferrochelatase, catalyzes ferrous insertion into protoporphyrin IX. (b) P16109, p-selectin, Ca^{2+}-dependent receptor, regulates the interaction between platelets and leukocytes. (c) O95989, diphosphoinositol polyphosphate phosphohydrolase 1, trans-element for ERK pathway. (d) P08397, porphobilinogen deaminase, catalyzes the reaction from porphobilinogen to hydroxymethylbilane. (e) P69905, hemoglobin subunit alpha, essential for oxygen transport. (f) Q9UNQ2, dimethyladenosine transferase, related to demethylation of two adjacent adenosines. (g) P08172, muscarinic acetylcholine receptor M2, regulates cellular responses.

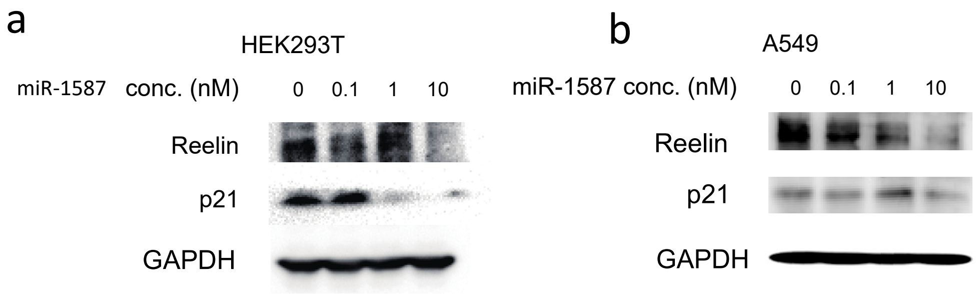

Figure S5. The Western blot assay to check CASK target genes Reelin and p21 expression in HEK293T cell line (a) and A549 cell line (b). Brief trend of Reelin/p21 was negatively regulated by miR-1587 could be noticed in all cell lines.

Table S1. 182 kinds of miR-1587 G-quadruplex binding proteins

Name	ID	SNR
ND	NM_138362.1	19.87
FTL	NM_000146.3	12.50
ACSL4	NM_022977.1	12.49
ZBTB2	ZBTB2	12.11
FFAR2	BC096201.1	10.55
CAMKK2	BC026060.2	10.45
TPM2	NM_003289.3	10.32
CPNE4	NM_130808.1	10.21
HNRNPC	BC008364.1	9.06
CASK	NM_001126054.	8.75
THYN1	BC093074.1	7.66
CPNE6	NM_006032.2	7.65
BZW2	NM_014038.1	7.52
RALYL	BC031090.1	7.39
GSN	BC026033.1	7.37
DCDC2	Q9UHG0	7.12
Supt6	BC072657	6.88
PYCR2	NM_013328.2	6.70
DCDC2	BC050704.1	6.15
NFKB2	BC002844.2	6.13
VWA8	NM_001009814.	6.10
ND	NM_017949.1	6.10
JMJD6	NM_015167.2	6.07
MMAB	NM_052845.2	6.02
EPB41	BC039079.1	5.98
HNRNPC	BC007052.1	5.93
TCOF1	NM_001008657.	5.90
ND	NM_004537.3	5.87
ZNF641	BC018090.1	5.76
MTERFD2	NM_182501.2	5.73
IMPDH1	P20839	5.72
ARL6IP4	BC001958.1	5.71
SDCBP	NM_001007069.	5.58
CAP1	NM_006367.2	5.56
RASGRP3	NM_170672.1	5.55
ZNF207	ID	5.54
SPSB2	NM_001032293.	5.51
MTERFD2	NM_032641.1	5.51
TAF7	BC053533.1	5.51

Name	NM_005642.2	SNR
LIMK2	NM_001031801.	5.48
FTL	BC016715.1	5.25
REEP6	NM_138393.1	5.19
USP7	NM_003470	5.16
EPS8L1	NM_139204.1	5.11
ASAP2	NM_003887.1	5.08
TIMELESS	NM_003920.2	5.08
CPNE2	NM_152727.4	4.98
C18orf54	BC036054.2	4.97
FTH1	BC066341.1	4.92
ACOT7	NM_181864.2	4.90
DNAJA4	BC031044.1	4.86
PALD1	NM_014431.1	4.86
RALY	BC103753.1	4.85
PSMA3	NM_002788.2	4.84
APIP	BC017594.2	4.78
ND	NM_004811.1	4.77
YBX3	BC009744.2	4.63
FTL	BC013928.1	4.62
THAP6	NM_144721.3	4.50
GARS	BC007722.2	4.46
EEF1G	BC013918.1	4.45
LUC7L	NM_018032.3	4.35
IMPDH2	NM_000884.2	4.34
ABI1	NM_001012752.	4.29
SPP1	BC093033.1	4.27
SSBP1	BC093054.1	4.27
UBE2D3	BC066917.1	4.26
TFAP2E	NM_178548.2	4.26
TPD52L3	NM_001001875.	4.23
TAF7	BC032737	4.22
ND	ND	4.21
HEPHL1	NM_001098672.	4.18
FAM109B	BC104176.1	4.16
PRKCDBP	ID	4.14
LARP4B	BC011585.1	4.12
VMA21	NM_015155.1	4.12
ZNF687	NM_001017980.	4.11
DCAF8	BC032463	4.10
LETM1	NM_015726.2	4.10

Name	NM_012318.1	SNR
GPS1	NM_212492.1	4.08
CLDN8	NM_199328.1	4.08
PSMA3	BC029402.1	4.04
LPL	BC011353.1	4.03
AIFM1	NM_004208.2	4.02
DMRT2	DMRT2	4.01
ND	NM_182684.1	3.99
MARCKSL1	BC066915.1	3.98
HNRNPU	BC015782.1	3.97
C11orf45	NM_145013.1	3.97
OPA3	BC047316.1	3.93
COLEC10	NM_006438.2	3.91
LACE1	NM_145315.3	3.88
CKM	BC007462.1	3.86
CATSPERD	BC043005.1	3.85
ZNF358	BC014002.1	3.83
FMO5	BC035687.1	3.80
MAGEA9	BC002351	3.80
VPS16	BC073959.1	3.79
SERPINE2	BC042628.1	3.79
PRRC2B	BC012289.1	3.78
PIP4K2A	NM_005028.3	3.75
SMIM11	NM_058182.3	3.75
ANKRD7	BC032799.1	3.72
PTGES3L-	NM_025267.2	3.70
PKM	BC035198.1	3.65
GSN	ENST000003412	3.65
XRCC4	NM_022406.1	3.64
ANKRD44	BC016985.2	3.64
C6orf141	BC036917.1	3.63
BEGAIN	NM_020836.2	3.62
42622	BC021192.2	3.60
CENPP	ID	3.59
LRRC6	NM_001012267.	3.58
FRK	BC027589.1	3.55
TFAP2D	NM_002031.2	3.54
SMN2	NM_172238.1	3.51
TEX264	NM_017411.2	3.51
VPS53	NM_015926.3	3.51
RTN4IP1	BC029560.1	3.51

Name	NM_032730.3	SNR
HSD17B2	NM_002153.1	3.50
IKZF1	XM_371901.1	3.50
SARS2	NM_017827.2	3.49
AKAP8L	NM_014371.2	3.49
TK1	BC007872.2	3.49
FAM115A	NM_014719.1	3.47
ZNF562	BC109062	3.46
PLA2G6	NM_001004426.	3.44
PACSIN1	NM_020804.2	3.42
EIF4B	BC098437.1	3.42
DNAJB5	NM_012266.3	3.41
RDX	BC047109.1	3.39
ZFP64	BC012759.2	3.38
KCNJ1	NM_153765.1	3.35
GSTA3	BC020619.1	3.35
HSCB	NM_172002.3	3.34
CYB5R3	NM_000398.4	3.34
ND	BC089417.1	3.34
SPATA20	NM_022827.2	3.34
MRPL1	BC015109.1	3.33
FGD5	BC035364.1	3.33
H1FX	NM_006026.2	3.33
MRPS28	NM_014018.2	3.32
MAK16	NM_032509.2	3.31
RPS6KA2	NM_021135.4	3.30
GSTM4	NM_000850.3	3.28
SRPX2	BC020733.1	3.27
CPSF4	NM_001081559.	3.27
CAST	NM_173060.2	3.26
PKNOX1	NM_004571.3	3.25
ND	ID	3.24
ST3GAL3	ENST000003617	3.24
ODF3L1	NM_006279.2	3.23
TCF7L1	NM_175881.2	3.22
IGHG1	TCF7L1	3.22
IGIP	BC092518.1	3.21
42621	NM_001007189.	3.20
TFDP3	BC001329.1	3.20
IRAK1BP1	BC117336	3.19
AGAP2	NM_001010844.	3.19

Name	BC028020.1	SNR
ZNF383	NM_152604.1	3.17
NAMPT	BC072439.1	3.17
UBE2N	NM_003348.3	3.13
PDXDC1	BC060871.1	3.13
RTN2	NM_206901.1	3.12
MIF4GD	NM_020679.2	3.12
MRTO4	NM_016183.2	3.11
DAZ2	BC113006	3.10
QKI	NM_206855.1	3.10
RPUSD3	NM_173659.2	3.10
OSBPL8	NM_001003712.	3.09
EPB41L3	BC006141.1	3.09
DAB1	BC067447.1	3.08
LPL	NM_000237.1	3.08
KDM1A	BC040194.2	3.08
EPB41L1	NM_177996.1	3.06
ADAMTSL4	BC027478.1	3.06
C1QL2	NM_182528.3	3.06
WISP2	BC058074.1	3.04
CNP	NM_033133.4	3.03
KIAA0020	BC016137.2	3.02
LRAT	NM_004744.3	3.02
PRM1	NM_002761.1	3.00

Table S2. 133 kinds of free form miR-1587 binding proteins

Name	ID	SNR
FTL	NM_000146.3	4.38
CCDC130	NM_030818.2	4.32
NAA16	NM_018527.2	3.72
BIRC5	BC008718.2	3.69
LPPR1	NM_017753.2	2.77
FTL	BC016715.1	2.65
PIK3R3	NM_003629.2	2.60
BAG6	NM_080702.1	2.57
COMMD10	NM_016144.2	2.54
ACAA1	NM_001607.2	2.50
FTL	BC021670.1	2.45
KDELC1	NM_024089.1	2.43
PPA2	NM_006903.4	2.39
ENPP7	BC041453.1	2.37
TIMM8B	NM_012459.1	2.33
MFSD8	NM_152778.1	2.32
OCIAD1	NM_017830.1	2.27
CCL16	NM_004590.2	2.27
ACSL4	NM_022977.1	2.26
FTH1	BC066341.1	2.23
RDH11	BC051291.1	2.21
PRRC2B	BC012289.1	2.20
ND	NM_004811.1	2.20
TMED5	BC038511.1	2.18
UQCRH	NM_006004.2	2.17
HMBS	NM_001024382.1	2.15
PYCR2	NM_013328.2	2.13
CASP2	BC002427.2	2.10
HMBS	NM_000190.3	2.08
ISY1	NM_020701.1	2.08
VHL	BC058831.1	2.01
FTL	BC013928.1	2.01
PHF23	NM_024297.2	2.01
RPL14	BC009294.2	2.01
BABAM1	NM_001033549.1	1.99
SPON1	NM_006108.3	1.98
hCG_1778643	BC035666.1	1.94

Name	ID	SNR
ND	NM_004284.2	1.93
IMPDH1	P20839	1.92
VMA21	NM_001017980.1	1.91
DCDC2	BC050704.1	1.91
IMPDH2	NM_000884.2	1.89
CCDC153	NM_001033658.1	1.88
FAM81A	NM_152450.1	1.86
PNPO	NM_018129.1	1.85
TXNRD1	NM_182729.1	1.85
LINC00311	BC030801.1	1.85
HLA-DQB1	BC012106.1	1.84
PKM	BC035198.1	1.80
FFAR2	BC096201.1	1.78
RPS12	NM_001016.3	1.78
ACRC	NM_052957.2	1.75
LIMK2	NM_001031801.1	1.74
PSMA3	NM_002788.2	1.72
PALD1	NM_014431.1	1.71
B3GALNT1	BC028571.1	1.70
IRAK1BP1	NM_001010844.1	1.69
CAMKK2	BC026060.2	1.68
WNT6	NM_006522.3	1.67
Blank	Blank	1.67
DNAJA4	BC031044.1	1.65
Blank	Blank	1.65
HEPHL1	NM_001098672.1	1.64
SPATA20	NM_022827.2	1.64
SPDL1	NM_017785.2	1.62
MAK16	BC039740.1	1.61
ND	NM_030593.1	1.60
BZW2	NM_014038.1	1.59
COMT	NM_007310.1	1.55
KRT8P41	BC093663.1	1.55
AIFM1	NM_004208.2	1.55
ATG16L1	ENST00000347464	1.54
CD3D	NM_000732.4	1.52
FBXO25	NM_012173.3	1.52
C18orf54	BC036054.2	1.52
ND	BC012848.1	1.51

Name	ID	SNR
IGHG1	BC006402.1	1.50
GSR	NM_000637.2	1.50
SULT4A1	BC030665.1	1.49
NKD1	NM_033119.3	1.47
HOXC5	NM_018953.2	1.47
ANXA6	NM_001155.3	1.46
EPS8L1	NM_139204.1	1.45
DTNB	BC049366.1	1.45
NFE2L3	NM_004289.5	1.45
SCCPDH	BC026185.2	1.43
ZRANB1	BC048281.1	1.43
MDM1	BC022042.1	1.42
MSRA	NM_012331.2	1.42
CAP1	NM_006367.2	1.42
PPFIBP2	NM_003621.1	1.42
SERPINE2	BC042628.1	1.42
NECAB1	BC016340.1	1.41
KLHL36	NM_024731.2	1.41
HDAC1	NM_004964.2	1.41
FAM114A1	BC001096.2	1.41
APITD1	NM_199295.1	1.40
ALDH1L1	BC027241.1	1.40
FRK	NM_002031.2	1.40
PWP1	BC001652.2	1.40
ACPP	NM_001099.2	1.40
LRP12	BC017381.1	1.40
MIF4GD	NM_020679.2	1.40
C6orf141	BC036917.1	1.40
TXNL4A	NM_006701.2	1.40
MIR4435-1HG	BC014776.1	1.39
ZFP64	BC012759.2	1.39
ZCCHC13	NM_203303.1	1.39
ND	NM_182684.1	1.38
LDB2	NM_001290.2	1.38
MPLKIP	NM_138701.1	1.37
SEPT8	BC001329.1	1.37
C11orf45	NM_145013.1	1.35
GYG2	BC023152.1	1.34
PSMC3	NM_002804.3	1.34

Name	ID	SNR
ST3GAL3	NM_006279.2	1.34
DNAJB2	NM_001039550.1	1.34
HN1	NM_016185.2	1.33
AP3S2	NM_005829.3	1.33
MAGEA9	BC002351	1.33
TBL3	BC035409.1	1.33
FAM115A	NM_014719.1	1.33
MMAB	NM_052845.2	1.31
C2orf15	NM_144706.2	1.31
MPP6	BC023638	1.31
Dmrt2	BC027669	1.30
LAS1L	BC019302.1	1.30
OR8B4	NM_001005196.1	1.30
CYP2U1	BC012027.1	1.30
HAO2	NM_001005783.1	1.30
SERTM1	NM_203451.1	1.30
SEMA6C	NM_030913.3	1.30
EFHC1	BC020210.1	1.30
